# A Rapid Progressive Submucosal Tumor Diagnosed as an Idiopathic Pseudoaneurysm in the Subserosal Gastric Wall: A Case Report

**DOI:** 10.70352/scrj.cr.26-0358

**Published:** 2026-07-25

**Authors:** Yuhei Nakano, Susumu Ohwada, Takayuki Aiba, Hideki Matsuyama, Atsushi Arakawa, Kinya Hayakawa

**Affiliations:** 1 Center of Gastroenterology and Oncology, IMS Ota Chuo General Hospital, Ota, Gunma, Japan; 2 Eil Inc. Itabashi Chuo Rinshoukensa Kenkyujo, Tokyo, Japan

**Keywords:** submucosal tumor, idiopathic arterial pseudoaneurysm, gastrectomy, gastric wall

## Abstract

**INTRODUCTION:**

The classic differential diagnosis of a progressive gastric submucosal tumor (SMT) includes gastrointestinal stromal tumor (GIST), lymphoma, and leiomyoma. But idiopathic pseudoaneurysm is seldom seen. We report the first case of a gastric idiopathic pseudoaneurysm that could not be diagnosed preoperatively.

**CASE PRESENTATION:**

An 83-years-old female was referred to our department (Center of Gastroenterology and Oncology, IMS Ota Chuo General Hospital) due to a 39 mm gastric mass accidentally found by CT scan. This mass had rapidly increased in size from 26 to 39 mm in 4 months. She had been seeing cardiology for atrial fibrillation on direct oral anticoagulant drug (DOAC) and chronic heart failure. Diffusion weight image (DWI) of MRI showed a high-intensity area in this mass. We diagnosed rapid progressive SMT and performed distal gastrectomy. The pathological finding was a pseudoaneurysm in the subserosal layer. Since she had no history of trauma or vasculitis, we diagnosed it as an idiopathic pseudoaneurysm. DOAC might contribute to the formation of this pseudoaneurysm. She had no recurrence in 3 years of follow up. To the best of our knowledge, this is the first reported case.

**CONCLUSIONS:**

Gastroenterologists should consider pseudoaneurysm in the differential diagnosis of rapid progressive SMT.

## Abbreviations

CECT
: contrast enhanced CT
DOAC
: direct oral anticoagulant drug
DWI
: diffusion weight image
eGFR
: estimated glomerular filtration rate
EUS
: endoscopic US
EUS-FNA
: endoscopic US guided fine needle aspiration
GIST
: gastrointestinal stromal tumor
SMT
: submucosal tumor
TAE
: transarterial embolization
T1WI
: T1-weighted imaging
T2WI
: T2-weighted imaging

## INTRODUCTION

The classic differential diagnoses of a progressive gastric SMT includes GIST, lymphoma, and leiomyoma. But idiopathic pseudoaneurysm is seldom seen. We report the first case of a gastric idiopathic pseudoaneurysm that could not be diagnosed preoperatively.

## CASE PRESENTATION

An 83-year-old female was referred to our department due to a 39 mm gastric mass accidentally found by a CT scan. This mass had rapidly increased in size from 26 to 39 mm in 4 months. She had been seeing a cardiologist for atrial fibrillation, chronic heart failure, and chronic kidney disease; and she had taken a DOAC for several years. She had no history of trauma or any gastrointestinal disease. She had no complaints. No abdominal mass was palpable.

Laboratory data were unremarkable except for chronic low hemoglobin (10.5g/dL), high blood urea nitrogen (29.7 mg/dL), creatinine (1.93 mg/dL) and eGFR (19.6 ml/min/1.73 m^2^); and soluble interleukin-2 receptor was relatively high (723 U/mL). Endoscopic examination revealed a 3 cm tumor located at the lesser curve of the antrum. The tumor was smooth and covered by normal gastric mucosa (**Fig. 1**). Biopsy was not performed. The CT scan demonstrated a 39 mm tumor protruding from the posterior wall. Comparison with previous CT scans showed no tumor at the same location 2 years earlier, whereas a 26 mm tumor was present 4 months earlier (**Fig. 2A**–**2C**). MRI showed a relatively high-intensity area within this mass. The borders of the tumor were well defined and its contents were heterogeneous and had a mosaic appearance on T2-weighted MRI (**Fig. 3A** and **3B**). In the DWI of MRI, its boundary had relatively high intensity (**Fig. 3C**). We diagnosed this as a rapidly progressive SMT, with an extra-luminally growing GIST being highly suspected. We planned an open partial gastrectomy without lymphoid dissection; however, limited distal gastrectomy was perfomed to facilitate anastomosis because the attachment surface between the tumor and gastric wall was much wider than expected and because this tumor was located 2 cm proximal to the pyloric ring on the lesser curvature. The tumor was relatively soft, smooth, and mobile. We did not perform lymphoid dissection because simple GIST with extra-luminal growth was still suspected through operative examination. There were no adhesions or bleeding. We adopted open surgery rather than a laparoscopic procedure to minimize the risk of iatrogenic rupture and to ensure specimen handling. A macroscopic view showed a subserosal tumor filled with hematoma when incised from the serosal side (**Fig. 4A** and **4B**). Before the incision, this hematoma was fully covered by a normal serosal wall. Pathologically, this hematoma was in a vascular wall-like structure and was located in the subserosal layer (**Fig. 4C**). Inflammatory cells infiltrated this arterial wall (**Fig. 4D**), and elastic fibers are detectable by elastic van Gieson staining (**Fig. 4E**). Since she had no history of trauma or vasculitis, we concluded that it was an idiopathic pseudoaneurysm. She had no recurrence in 3 years of follow up. And this is the first report case to the best of our knowledge.

**Fig. 1:**
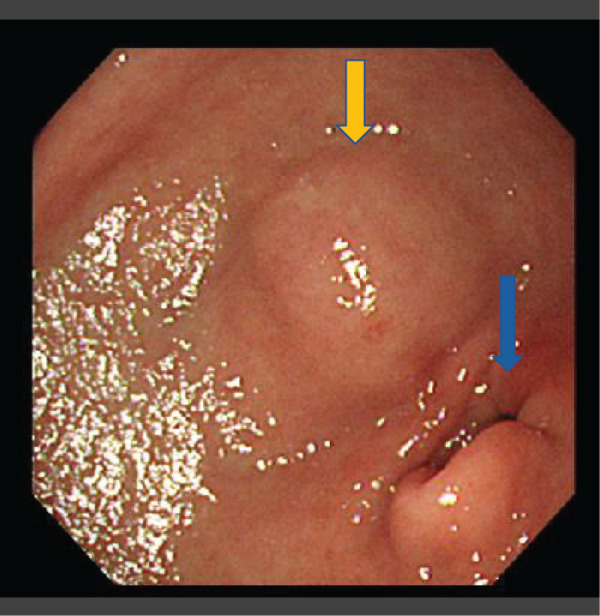
Endoscopic findings. Endoscopy showed a 3 cm tumor located at the lesser curvature of the antrum (yellow arrow). Pyloric ring (blue arrow).

**Fig. 2:**
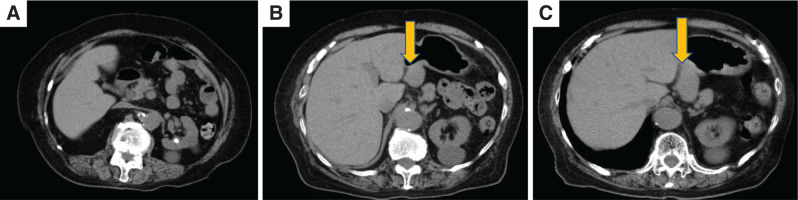
CT. (**A**) No tumor was seen 2 years earlier. (**B**) A 26 mm tumor was seen. 4 months earlier (arrow). (**C**) A 39 mm tumor was protruding from the posterior wall at the time of referral (arrow).

**Fig. 3:**
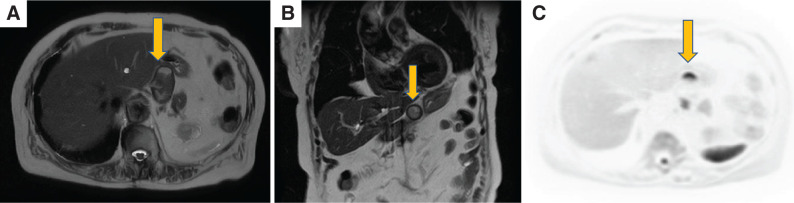
MRI. (**A**) T2 enhanced MRI, axial view. Relatively high intensity area on lower gastric wall. The border of the tumor was clear and its contents were heterogeneous and mosaic (arrow). (**B**) T2 enhanced MRI, coronal view. Relatively high intensity area on lower gastric wall. The border of the tumor was clear and its contents were heterogeneous and mosaic (arrow). (**C**) In the diffusion weight image of MRI, its border was relatively high intensity (arrow).

**Fig. 4:**
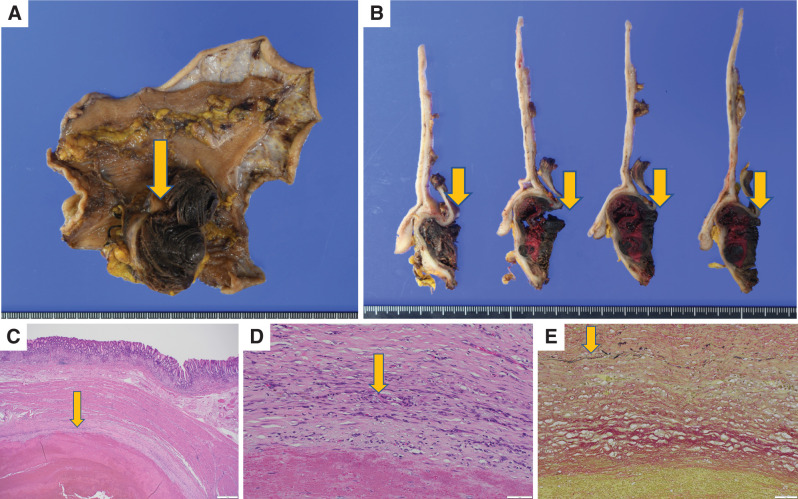
Macroscopic and pathological findings. (**A**) Macroscopically, when incised from the serosal side, a 35 mm hematoma was observed (arrow). The hematoma was cut from the serosal side. (**B**) Macroscopic cross-sectional view. A 35 mm hematoma was observed (arrows). The hematoma was cut from the serosal side. (**C**) Pathologically, this hematoma was in a vascular wall-like structure and was located in the subserosal layer (arrow). (**D**) Inflammatory cells infiltrated this arterial wall (arrow). (**E**) Elastic fibers were detectable by elastic van Gieson staining (arrow).

## DISCUSSION

Because of her poor renal function, we could not use contrast media, so MRI was performed. The diagnosis of gastric GIST is sometimes challenging because of the diverse imaging findings, such as the growth pattern and the enhancement pattern, as well as the presence of necrosis, calcification, and ulceration. The density of GIST on noncontrast CT is similar to that of the muscles, and its contrast effect varies. The morphological features visualized on CT enable the prediction of high-grade GIST and poor prognosis. Generally, the growth pattern of GIST is categorized as exophytic, intraluminal, and mixed/combined/endophytic. In gastric GIST, exophytic and mixed growth patterns are correlated with high-grade GIST and high mitotic counts. Necrosis appears as a cystic component on CT and MRI. Intratumoral hemorrhage or necrosis develops when the hypervascular tumor outgrows its blood supply, which is associated with a heterogeneous tumor texture. On MRI, GIST typically show low signal intensity on T1WI and high signal intensity on T2WI. Additionally, the apparent diffusion coefficient obtained by DWI is helpful in assessing malignancy.^1)^ In the present case, a tumor with an exophytic growth pattern was seen, and its contents were heterogeneous with a mosaic appearance on T2-weighted MRI, implying inratumoral hemorrhage or necrosis mimicking malignant GIST. In contrast, pseudoaneurysms consistently showed void morphology and central flow void on both T1WI and T2WI. Neurovascular bundle involvement can also be observed.^2)^ In the present case, none of the above signs were detected. The reason was believed to be that the damaged vessel was a microvessel in the gastric wall. Since the blood flow itself is likely slower than that in medium-sized or large arteries such as femoral and splenic arteries, the difference in flow velocity between the center and periphery would also be small. The neurovascular bundles were also too small to be detected. If CECT had been performed, pooling of contrast media, which might suggest the possibility of a pseudoaneurysm, could have been detected.

We performed surgery without EUS and EUS-FNA, because the malignant features such as rapid growth and its heterogeneous contents were obvious. According to Japanese guidelines for GIST, if an SMT larger than 2 cm exhibits malignant features such as those described above, surgery or biopsy/excision is recommended, regardless of whether EUS is performed. This guideline is very similar to those of the National Comprehensive Cancer Network and the European Society for Medical Oncology.^3)^ If EUS had been performed, this tumor would have been seen in the 5th layer, and color Doppler imaging might have provided further information such as blood flow. If an arterial pseudoaneurysm is suspected, we must avoid EUS-FNA. This tumor grew by 13 mm in 4 months, meaning a doubling time of approximately 10 months. If we consider the mean doubling time of GIST to be 377 days,^4)^ this growth rate is rapid. As a result, the tumor was filled with hematoma, and we concluded that this progression was due to bleeding. Given that this pseudoaneurysm was located in the sub-serosal layer, we assumed that a microvessel in this layer had somehow been damaged, leading to the formation of the pseudoaneurysm. Retrospectively, no other aneurysms were seen on CT scan.

A pseudoaneurysm is a bulge filled with hematoma following an arterial wall rupture. Therefore, it lacks the 3-layered structure of the arterial wall and is covered only by connective tissue or arterial adventitia. In the present case, the wall was composed of the connective tissue forming an artery-like structure. The wall is fragile and its rupture can lead to serious complications. For this reason, if a pseudoaneurysm is definitively diagnosed, resection or TAE is recommended.^5)^ Since TAE for gastric intramural haematomas or bleeding has already been reported,^6)^ TAE can be recommended for gastric pseudoaneurysm.

Gastric subserosal tumor is rare. Apart from intraluminal abscesses, the only reported gastric subserosal tumors were lipoma^7)^ and liposarcoma.^8)^ To the best of our knowledge, there is no report of idiopathic gastric pseudoaneurysm. Since pathological findings showed inflammatory cell infiltration and elastic fibers in the arterial wall, an inflammatory reaction could potentially contribute to the pseudoaneurysm. The possible causes are intraluminal abscess, gastric ulcer, and injury with fishbone, although the surface of the mucosa was normal. Since pseudoaneurysms as a complication of anticoagulation therapy have been reported, it is possible that DOAC contributed to forming the pseudoaneurysm.^9,10)^

No specific recommendations exist for follow up or surveillance for visceral pseudoaneurysms. For embolized gastroepiploic lesions, recommendations for surveillance include imaging every 1–2 years; however, no recommendations exist for surveillance following resection of these lesions.^9)^ However, because the etiology is still unclear, there is a possibility that pseudoaneurysms may form in other locations in the future. Therefore we followed up by annual CT scan and no recurrence was seen in 3 years.

## CONCLUSIONS

We report the first case of a rapid progressive SMT diagnosed as an idiopathic subserosal arterial pseudoaneurysm in a gastric wall. Clinicians should consider pseudoaneurysm in the differential diagnosis of rapidly progressive SMT and avoid EUS-FNA if a pseudoaneurysm suspected. If a pseudoaneurysm is confirmed, resection or TAE is recommended.

## DECLARATIONS

### Use of artificial intelligence tools

No artificial intelligence tool was used.

### Availability of data and materials

Data sharing is not applicable for this article as no datasets were generated or analyzed during the current study.
